# Is increasing the effective leg length of a human runner metabolically beneficial?

**DOI:** 10.1242/jeb.250107

**Published:** 2025-10-22

**Authors:** Montgomery Bertschy, Herlandt Lino, Laura Healey, Wouter Hoogkamer

**Affiliations:** ^1^ Integrative Locomotion Laboratory, Department of Kinesiology, University of Massachusetts, Amherst, MA 01003, USA; ^2^ PUMA SE, Innovation, Somerville, MA 02155, USA

**Keywords:** Footwear, Running, Metabolic cost, Midsole thickness, Advanced footwear technology

## Abstract

The purpose of this study was to understand whether footwear midsole thickness can decrease the metabolic cost of running by increasing the effective leg length of a runner. We also sought to understand the effect of midsole compliance on these measures. Participants (*N*=16) ran on a treadmill at 14 km h^−1^ in four mass-matched shoe conditions: 30, 40 and 60 mm thickness of a firm midsole foam and 60 mm thickness of a compliant midsole foam. Over two testing sessions, we measured metabolic cost (two 5-min trials in each condition) and biomechanical measures (one 2-min trial in each condition). Increasing thickness in the firm midsole conditions increased effective leg length during early, mid and late stance (all *P*<0.001). However, metabolic cost increased (*P*=0.031). Changing material, from firm to compliant, decreased effective leg length during midstance (*P*<0.001), but not during early or late stance. This change in material also decreased metabolic cost (*P*<0.001). Results suggest that increasing midsole thickness can increase the effective leg length of a runner, but this isolated effect leads to increases in metabolic cost. In contrast, increasing midsole compliance, while keeping midsole thickness constant, leads to reductions in metabolic cost, agreeing with previous literature. We suggest that the performance benefits seen in advanced footwear technology are likely due to increased resilience of the midsole but not to increases in effective leg length from increased midsole thickness.

## Teaser

**Summary:** Increased midsole resilience may be responsible for decreased metabolic cost of running in footwear with high midsole thickness, not increased effective leg length.

## INTRODUCTION

Running shoes have been increasing in thickness since the introduction of advanced footwear technology (AFT) in 2016 (Joubert and Jones, 2022). AFT is defined as performance running shoes with a highly compliant and resilient midsole, embedded stiffening elements, and a geometry that has a greater midsole thickness and more pronounced rocker than traditional running footwear (Frederick, 2022). Since the introduction of AFT, significant improvements in elite road racing performances in nearly every distance have occurred (Bermon et al., 2021; Mason et al., 2024; Senefeld et al., 2021). Such improvements have resulted in controversy over footwear technology, resulting in the regulation of midsole thickness by World Athletics in 2020. These regulations were made based on the idea that increasing the thickness of footwear can increase the effective leg length of the runner, improving their performance (Burns and Tam, 2020).

Across the animal kingdom, greater effective limb length (i.e. hip height) is correlated with lower metabolic cost of transport, outperforming both contact time and body mass as predictors (Pontzer, 2005, 2007a,b). Smaller animals have a higher rate of muscular force production (i.e. shorter contact time) to support their body mass during locomotion than larger animals owing to them taking shorter, more frequent steps, resulting in a higher mass-specific cost of transport (Kram and Taylor, 1990). Animals with longer limb length take longer, less frequent steps (Kram and Taylor, 1990), and longer limb length is highly correlated with longer contact time (Hoyt et al., 2000). A model incorporating effective limb length and simple kinematic variables is able to predict the cost of transport within species better than contact time by taking into account both the cost to support body mass and the cost to swing the limb (Pontzer, 2005, 2007b). However, it is not well understood whether the effective limb length within an individual can be modified to gain energetically efficient locomotion. A potential method to increase effective leg length in humans is footwear (Barrons et al., 2023; Burns and Tam, 2020; Mason et al., 2023, 2024). Theoretically, by increasing leg length with footwear of high midsole thickness without adding additional physiologically costly tissue, the runner may increase their stride length with minimal additional metabolic cost, thereby reducing the metabolic cost of running. Additionally, because females are, on average, shorter in stature than males, the relative contribution of a given midsole thickness to increased effective leg length may be larger, which may coincide with females showing greater improvements in race times since the introduction of AFT (Mason et al., 2024). However, a longer effective leg length may not be entirely beneficial. If stride length increases from a longer effective leg length, the moment arm length of the horizontal component of the ground reaction force about the hip may increase, theoretically increasing the sagittal hip extension moment and increasing metabolic demands of the hip extensor muscles.

On the other hand, midsole thickness may play a much different role in running metabolic cost. Midsoles in AFT are compliant, resulting in greater midsole deformation under the same load than traditional footwear, often needing greater thickness to prevent bottoming out the midsole (the phenomenon whereby the midsole stiffens up exponentially when the deformation approaches the overall thickness) (Hoogkamer et al., 2018; Kram, 2022; Shorten, 2024). These midsole materials also tend to be highly resilient, showing high energy return in mechanical testing, but the effect of midsole energy return on the runner is highly debated (Kram, 2022; Matijevich et al., 2023; Shorten, 2024). Several studies have shown that softer midsole materials tend to reduce running metabolic cost (Frederick et al., 1986; Rodrigo-Carranza et al., 2024; Worobets et al., 2014), but there is also evidence that more underfoot cushioning does not always lead to improvements in metabolic cost (Hoogkamer, 2020; Tung et al., 2014).

Because more compliant midsole materials will lead to greater midsole compression, it is reasonable to assume that a firmer midsole material will be more likely to extend effective leg length throughout stance. Therefore, if the metabolic cost of running is decreased by increasing the midsole thickness with a firm material, then increased effective leg length may be a mechanism for reducing running metabolic cost in AFT. However, if the metabolic cost of running is decreased by increasing the compliance of a thick midsole, then increased energy absorption (and potentially return) may be a mechanism for improved running metabolic cost in AFT. The purpose of this study was to test the isolated effects of midsole thickness on running metabolic cost and biomechanics. The first aim was to test the concept of increased effective leg length as a mechanism for reducing the metabolic cost of running. We hypothesized that increased midsole thickness would lead to a reduced metabolic cost while running by increasing the effective leg length of the runner. The second aim was to test how midsole properties contribute to the effect of midsole thickness on the metabolic cost of running. We hypothesized that greater midsole compliance and resilience would lead to a reduced metabolic cost while running. Additionally, we quantified joint mechanics to provide future insights into the effects of midsole properties on running biomechanics and energetics.

## MATERIALS AND METHODS

### Participants

The study protocol was approved by the University of Massachusetts institutional review board (IRB #2927). We recruited runners 18–45 years of age that were capable of running a 5-km race within 17 min 30 s. Participants were free of musculoskeletal injury at the time of participation and did not have surgery within 6 months of data collection. Eighteen (13 male and five female) runners participated in the study. Two participants were unable to maintain a respiratory exchange ratio below 1.0 during metabolic testing and were removed from analysis. We analyzed data for the remaining 16 participants (12 males and four females; age 25.4±7.2 years; height 175.6±8.8 cm; mass 65.3±8.0 kg; standing leg length 92.6±5.2 cm).

### Footwear conditions

**Fig. 1.**
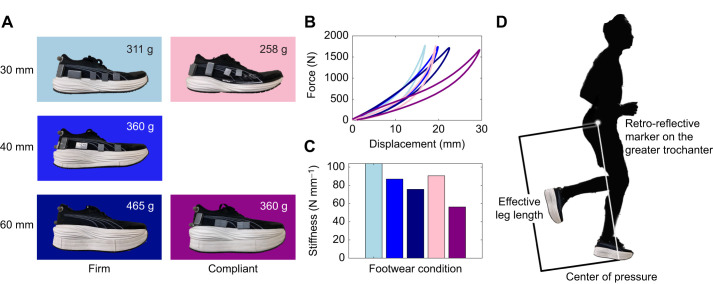
**Summary of experimental footwear conditions.** (A) Footwear conditions with midsole thicknesses of 30, 40 and 60 mm constructed with a firm material (shades of blue), and footwear conditions with midsole thicknesses of 30 and 60 mm constructed with a compliant material (shades of pink). All footwear conditions were mass-matched to the heaviest condition (60 mm firm, 465 g). (B,C) Plots of force versus displacement (B) and compression stiffness (C) from mechanical testing of each footwear condition. Colors used in B and C match the colors surrounding the respective footwear conditions in A. (D) Effective leg length was calculated as the sagittal plane distance from the greater trochanter to the center of pressure.

We used five footwear conditions in this study: shoes with midsole thicknesses of 30 mm, 40 mm and 60 mm, made from firm ethylene-vinyl acetate (EVA) foam (Asker C hardness 52±2); and shoes with a midsole thickness of 30 and 60 mm, made from a highly compliant and resilient polyether block amide (PEBA)-based material (Asker C hardness 43±2). All footwear conditions were custom made based on the commercially available PUMA Deviate Nitro (30 mm compliant; see Fig. 1) by adding additional layers of foam between the midsole and outsole, keeping embedded stiffening plate placement consistent relative to the top of the midsole (15.9 mm in the forefoot and 5 mm in the rear foot). All footwear conditions were matched for mass based on the heaviest condition (60 mm firm, 465 g) by adding steel masses (∼7 or 28 g each) distributed along the rear 75% of the shoe, glued to the base of the upper (Perry et al., 2025). Part 1 of the study included 30, 40 and 60 mm firm and 60 mm compliant conditions. Part 2 of the study included 30 mm firm and 30 and 60 mm compliant conditions.

### Mechanical testing

The footwear compression stiffness, energy input, output and percentage return were measured using a material testing machine (Instron ElectroPuls 10000, Instron, Norwood, MA, USA). A rounded probe (diameter 43 mm; radius of curvature 21.5 mm) applied a parabolic force profile with a peak magnitude of 1800 N for 185 ms onto the heel of the shoe. Midsole compression stiffness was calculated from the maximum force applied and the maximum displacement (Table 1). Energy return was calculated as the ratio from the area under the force-deformation curve during unloading (output energy) to loading (input energy) (Table 1).

**Table 1.** Summary of mechanical testing results of footwear conditions

Thickness (mm)	Material	Input energy (J)	Output energy (J)	Energy return (%)	Compression stiffness (N mm^−1^)	Asker C hardness
30	Firm	9.8	7.0	71.9	104.0	52±2
40	Firm	13.7	9.9	72.1	86.8	52±2
60	Firm	17.3	12.3	71.1	75.6	52±2
30	Compliant	10.1	7.7	76.0	90.4	43±2
60	Compliant	20.5	15.3	74.5	56.2	43±2

### Experimental protocol

Participants attended two data collection sessions: a metabolic session and a biomechanics session. The metabolic session consisted of a self-paced running warm up of at least 5 min in their own shoes. Next, for familiarization, participants ran 5 min in their own shoes on a force-instrumented, rigid treadmill (Treadmetrix, Park City, UT, USA) at the experimental speed of 14 km h^−1^ while breathing into a mouthpiece for indirect calorimetry. Participants then ran in each of the four shoe conditions (firm, 30, 40, 60 mm; compliant, 60 mm) twice in randomized, mirrored order (ABCDDCBA) for 5 min at 14 km h^−1^, followed by 5 min rest, for a total of eight running trials, while we measured expired air (TrueOne 2400, ParvoMedics, Salt Lake City, UT, USA). The expired gas analysis system was calibrated before the first (ABCD) and second (DCBA) halves to reduce long-term analyzer drift. The outcome measure for this session was metabolic cost of running calculated as metabolic power (W kg^−1^).

The biomechanics session consisted of a self-paced running warm up of at least 5 min. Next, we placed 21 retro-reflective markers on the shoe tip, first and fifth metatarsal head, medial and lateral malleolus, medial and lateral epicondyle, greater trochanter, and left and right anterior and posterior superior iliac spine. Additional marker clusters were placed on the heel, shank and thigh. Participants ran in each shoe condition in randomized order for 2 min while we measured kinematics (Oqus, Qualisys, Goteborg, Sweden) and ground reaction forces (GRFs) (Treadmetrix, Park City, UT, USA). The outcome measures for this experiment were leg length during stance (m), total and vertical stiffness of the leg plus midsole (N mm^−1^), GRFs (N), step length (m), and joint angles (deg), moments (Nm) and powers (W kg^−1^). There was no minimum duration between the two data collection sessions, as fatigue was minimal (e.g. four participants completed the sessions directly after one another, whereas 12 participants completed the two sessions on separate days).

We performed additional exploratory measurements with separate participants and a 30 mm compliant condition in an attempt to separate out the effects of thickness and material properties on the differences between the 30 mm firm and 60 mm compliant conditions (six male; age 20.8±3.1 years; height 176.0±7.3 cm; mass 68.5±5.5 kg). Although we cannot make any strong claims, as these data lack statistical power, we wish to present these data for the reader. This metabolic session consisted of a self-paced running warm up of at least 5 min in their own shoes. Next, for familiarization, participants ran 5 min in their own shoes on a force-instrumented, rigid treadmill at the experimental speed of 14 km h^−1^ while breathing into a mouthpiece for indirect calorimetry. Participants then ran in each of the three shoe conditions (firm, 30 mm; compliant, 30, 60 mm) twice in randomized, mirrored order (ABCCBA) for 5 min at 14 km h^−1^, followed by 5 min rest, for a total of six running trials, while we measured expired air. The outcome measure for this session was metabolic cost of running calculated as metabolic power (W kg^−1^).

### Data analysis

We calculated metabolic power based on the rates of oxygen uptake and carbon dioxide production over the final 2 min of each trial (Péronnet and Massicotte, 1991). GRF data were collected at 1000 Hz and lowpass filtered with a second-order dual-pass Butterworth filter at 15 Hz. Heel strike was defined as the time when vertical GRF increased above 30 N, midstance as the time of peak vertical GRF, and toe off as the time when vertical GRF decreased below 30 N. Step length was calculated as the time from heel strike to heel strike of the opposite foot multiplied by the treadmill velocity. Contact time was time from heel strike to toe off. Standing leg length was determined as the vertical distance from the greater trochanter to the ground during the static trial in the 30 mm firm condition. Effective leg length during stance was determined as the distance from the greater trochanter to the center of pressure during early stance (10% of stance), midstance and late stance (90% of stance) (excluding the first and last portion of stance to remove extreme values of center of pressure location related to noise during the aerial phase and filtering artefacts) (Hoogkamer et al., 2019; Willwacher et al., 2014). We defined leg stiffness as the peak vertical GRF divided by the center of mass (estimated as the mean locations of the left and right anterior and posterior superior iliac spine) displacement in the vertical and anterior–posterior direction (relative to the center of pressure) from heel strike to midstance (Arampatzis et al., 1999). We defined vertical stiffness as the peak vertical GRF divided by the center of mass displacement in the vertical direction from heel strike to midstance. Leg stiffness and vertical stiffness calculations include leg plus midsole length changes. Joint mechanics were processed in Visual 3D (C-Motion, Germantown, MD, USA) based on a 6-degrees-of-freedom model.

### Statistical analysis

We used linear mixed effect models (Wilkinson et al., 2023) in our analysis using the basic formula:
(1)

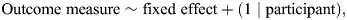
where the fixed effect is either midsole thickness (30, 40, 60 mm firm material) or material (firm and compliant at 60 mm). Effect sizes were calculated as partial eta squared (η^2^), which indicates the proportion of the variance of the outcome measure explained by the fixed effect. η^2^ values of 0.01, 0.06 and 0.14 indicate small, medium and large effects, respectively (Richardson, 2011). All previously described outcome measures were tested across midsole thickness and material at relevant timepoints (early, mid and late stance for leg length and vertical/leg stiffness; peak for joint angle, moment and power). We performed all statistical analyses in R (R software version 2022.12.0.353).

### Artificial intelligence

R, Python and MATLAB code for data analysis and figures were created with some assistance from ChatGPT (OpenAI, 2024).

## RESULTS

### Midsole thickness

**Fig. 2.**
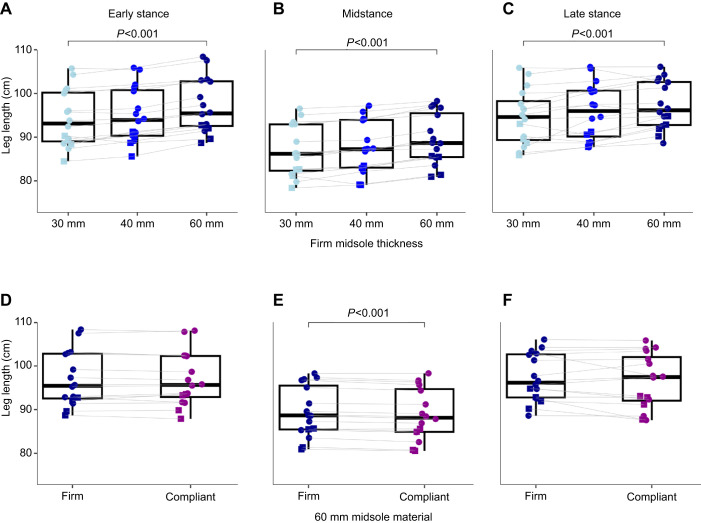
**Leg length increases at heel strike, midstance and toe off as midsole thickness increases; leg length decreases during midstance when midsole material is more compliant.** (A–C) Leg length during early, mid and late stance across firm midsole thicknesses. (D–F) Leg length during early, mid and late stance in both 60 mm midsole materials. Male and female participants are denoted by circles and squares, respectively. Box plots show medians, upper and lower quartiles range. Whiskers show ±1.5× interquartile ranges.

Increasing thickness of the firm midsole material led to an increased leg length during early stance (*P*<0.001, η^2^=0.797), midstance (*P*<0.001, η^2^=0.830) and late stance (*P*<0.001, η^2^=0.420) (Fig. 2A–C, Table 2). Vertical stiffness was reduced as midsole thickness increased (*P*=0.018, η^2^=0.166), but leg stiffness was unchanged.

**Table 2.** Summary of biomechanical measures

	Midsole condition				Thickness effect		Material effect	
	30 mm firm	40 mm firm	60 mm firm	60 mm compliant	*P*-value	η^2^	*P*-value	η^2^
Leg length during early stance (cm)	94.2±6.5	95.1±6.3	97.0±6.3	97.0±6.1	<0.001	0.797	0.676	0.012
Leg length during midstance (cm)	86.9±5.8	87.7±5.9	89.5±5.8	89.0±5.8	<0.001	0.830	<0.001	0.745
Leg length during late stance (cm)	94.6±6.3	95.9±6.4	97.3±5.5	96.6±6.3	<0.001	0.420	0.239	0.091
Leg stiffness (N mm^−1^)	24.4±4.1	24.9±6.9	23.6±5.1	22.2±4.8	0.398	0.023	0.006	0.405
Vertical stiffness (N mm^−1^)	37.1±5.4	36.8±5.9	36.2±5.3	35.6±5.3	0.018	0.166	0.178	0.117
Contact time (s)	0.217±0.012	0.217±0.012	0.218±0.014	0.220±0.013	0.159	0.063	0.038	0.258
Step length (m)	1.33±0.07	1.33±0.07	1.33±0.07	1.33±0.07	0.594	0.009	0.693	0.011
Peak vGRF (N)	1754±276	1749±262	1735±272	1722±260	0.085	0.093	0.169	0.122
Braking/propulsive impulse (Ns)	43.8±7.5	43.1±7.1	42.5±7.0	44.2±7.3	<0.001	0.398	<0.001	0.748
Metabolic power (W kg^−1^)	16.09±1.04	16.20±1.02	16.27±0.93	15.81±0.93	0.031	0.623	<0.001	0.387

Values reported as means±s.d. vGRF, vertical ground reaction force.

**Fig. 3.**
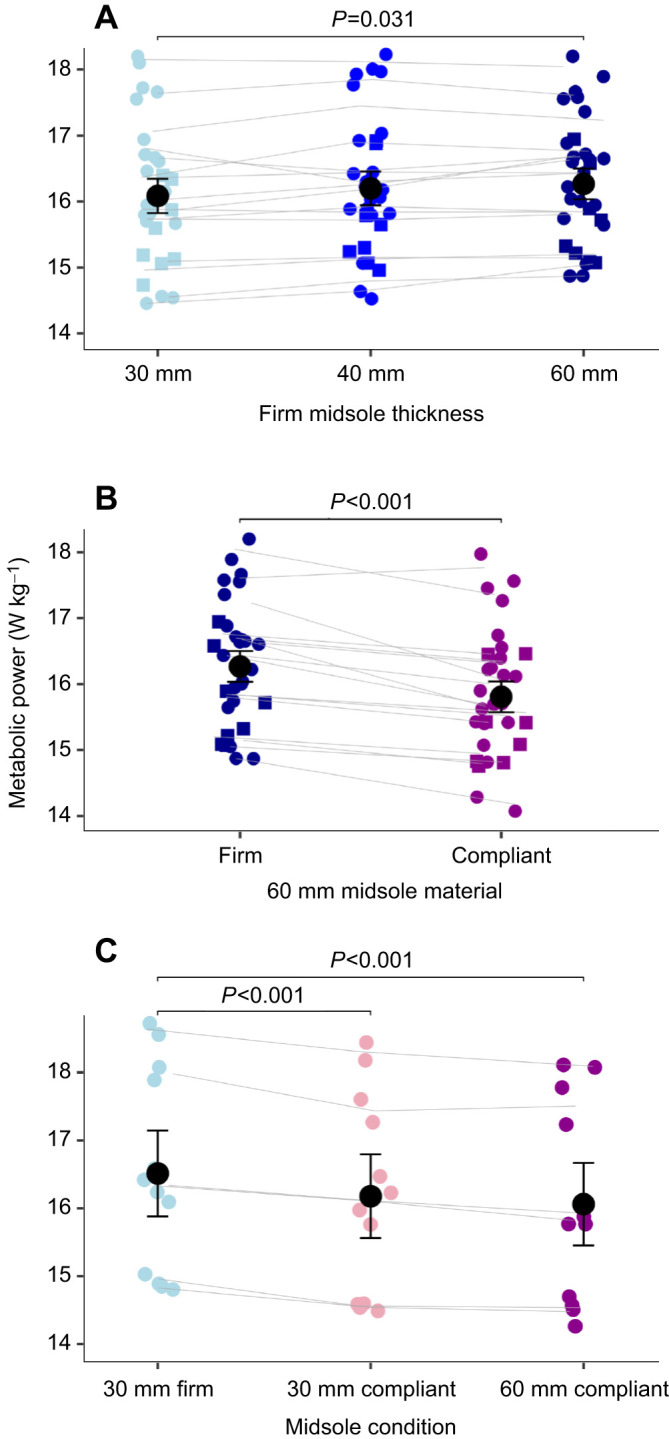
**Metabolic power increased with increasing midsole thickness but decreased when midsole material was more compliant.** (A,B) Effects of firm midsole thickness (A) and 60 mm midsole material (B) on metabolic power in part 1 of the study (*N*=16). Male and female participants are denoted by circles and squares, respectively. Gray lines represent participant mean; colored points represent each trial. (C) For the exploratory findings (*N*=6), all conditions are presented. Values are reported as mean±s.e.m.

Increasing thickness of a firm midsole material led to a 0.4% increase in metabolic power per 10 mm of added material (*P*=0.031, η^2^=0.623) (Fig. 3A). There was no effect of midsole thickness on step length, contact time or peak vertical GRF. Braking and propulsive impulses decreased with increased midsole thickness (*P*<0.001, η^2^=0.398).

**Fig. 4.**
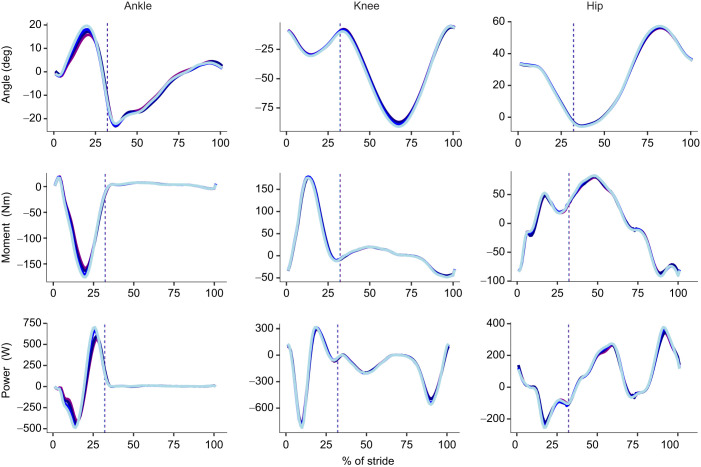
**Summary of sagittal joint kinematics and kinetics.** Results on angle (top row), moment (middle row) and power (bottom row) and percentage of stride for the ankle (left column), knee (middle column) and hip (right column). Solid lines represent the mean of the respective sagittal plane variable for 30 mm firm (light blue), 40 mm firm (blue), 60 mm firm (dark blue) and 60 mm compliant (purple) conditions. Vertical dashed lines represent the end of stance.

**Fig. 5.**
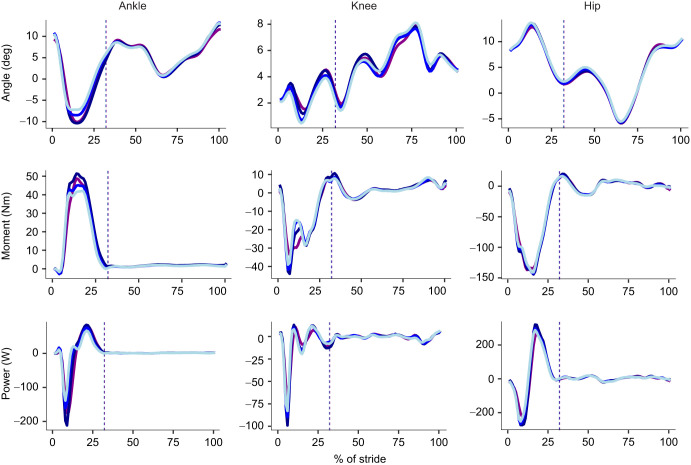
**Summary of frontal joint kinematics and kinetics.** Results on angle (top row), moment (middle row) and power (bottom row) and percentage of stride for the ankle (left column), knee (middle column) and hip (right column). Solid lines represent the mean of the respective frontal plane variable for 30 mm firm (light blue), 40 mm firm (blue), 60 mm firm (dark blue) and 60 mm compliant (purple) conditions. Vertical dashed lines represent the end of stance.

In the sagittal plane (Fig. 4), increasing thickness of the firm midsole material led to a decrease in peak ankle dorsiflexion angle (*P*<0.001, η^2^=0.547), plantarflexion moment (*P*<0.001, η^2^=0.493), positive (*P*<0.001, η^2^=0.652) and negative power (*P*<0.001, η^2^=0.383); a decrease in peak knee flexion angle (*P*=0.008, η^2^=0.139) and positive power (*P*=0.002, η^2^=0.183); a decrease in peak positive hip moment (*P*=0.020, η^2^=0.110) and peak negative power (*P*=0.038, η^2^=0.088); and an increase in hip positive power (*P*<0.001, η^2^=0.227) at heel strike. In the frontal plane (Fig. 5), increasing thickness of the firm midsole material led to an increase in peak ankle eversion angle (*P*<0.001, η^2^=0.261), eversion moment (*P*=0.028, η^2^=0.099), and positive (*P*=0.033, η^2^=0.093) and negative power (*P*<0.001, η^2^=0.295), but no differences at the knee or hip.

### Midsole material

At the largest midsole thickness, the compliant material led to a significantly lower leg length at midstance (*P*<0.001, η^2^=0.745) but not during early or late stance (Fig. 2D–F, Table 2). Leg stiffness was significantly reduced with a firm compared with a compliant midsole material (*P*=0.006, η^2^=0.405). Vertical stiffness was not different across midsole material.

At the 60 mm midsole thickness, the compliant material led to a 1.7% lower metabolic power than the firm material (*P*<0.001, η^2^=0.387) (Fig. 3B). Contact time was larger with a firm compared with a compliant midsole material (*P*=0.038, η^2^=0.258), but there were no significant changes in step length or peak vertical GRF with midsole material. Braking and propulsive impulses were larger with a firm compared with a compliant material (*P*<0.001, η^2^=0.748).

In the sagittal plane (Fig. 4), at the largest midsole thickness, compared with the firm material, the compliant material led to a significantly reduced peak ankle dorsiflexion angle (*P*=0.037, η^2^=0.259), plantarflexion moment (*P*=0.002, η^2^=0.471), and positive (*P*=0.002, η^2^=0.485) and negative power (*P*=0.0165, η^2^=0.327), but no differences in knee and hip mechanics. In the frontal plane (Fig. 5), compared with the firm material, the compliant material led to a significantly reduced peak ankle eversion moment (*P*=0.012, η^2^=0.352) and negative power (*P*=0.023, η^2^=0.301); reduced peak adduction knee moment (*P*=0.004, η^2^=0.434) and negative power (*P*=0.006, η^2^=0.410); and reduced peak adduction hip angle (*P*<0.001, η^2^=0.544), abduction moment (*P*=0.010, η^2^=0.367), and positive (*P*=0.001, η^2^=0.525) and negative power (*P*=0.006, η^2^=0.411).

### Exploratory findings

At the 30 mm midsole thickness, the compliant material led to a 2.4% lower metabolic power than the firm material (*P*<0.001, η^2^=0.538) (Fig. 3C). However, we did not find a significant difference between the 30 and 60 mm compliant midsole conditions, and there was no clear trend that could lead to speculation on the effect of midsole thickness of compliant material on metabolic cost, which is to be expected with our relatively small sample size.

## DISCUSSION

### Midsole thickness

This study sought to determine whether increasing the effective leg length of a runner reduces the metabolic cost of running. We found that effective leg length was increased at early, mid and late stance as midsole thickness increased, achieving our goal for experimental conditions. This is in agreement with existing literature that has found midsole thickness to be associated with effective leg length (Barrons et al., 2023). Despite running with an increased effective leg length, participants did not increase their step length. In overground running, step length can be increased while maintaining step frequency, resulting in increased speed. Here, participants ran at a fixed speed on a treadmill, so increasing step length would require a decrease in step frequency, which may not be advantageous for metabolic cost as it can result in suboptimal muscle–tendon dynamics (Swinnen et al., 2022). Yet, other studies have seen increases in step length in different footwear conditions that led to favorable metabolic outcomes (Franz et al., 2012; Hoogkamer et al., 2018; Hunter et al., 2019; Rodrigo-Carranza et al., 2024). It may be that speeds higher than 14 km h^−1^ are needed to elicit changes in step length, as increases in step length have been seen in sprinting in taller shoes (Mason et al., 2023), and transtibial amputees have also achieved greater step lengths with greater prosthetic length (Beck et al., 2017). However, as discussed later, changes in frontal plane mechanics with increased midsole thickness may reduce the stability of the runner, hindering the use of longer strides.

Contrary to our hypothesis, as effective leg length increased, metabolic cost did not decrease, but increased. Although the relationship between leg length and metabolic cost of transport is strong across the animal kingdom (Pontzer, 2007a), this does not appear to be the case within individual humans. At similar speeds, there is some evidence that humans with longer legs have a lower metabolic cost than those with shorter legs (Brisswalter et al., 1996), but these findings do not transfer to our present results within individuals. These findings also diverge from the findings of Barrons et al. (2023), who reported no difference in metabolic cost across midsole thickness. There are several distinctions to be made regarding the footwear conditions used in Barrons et al. (2023) and our study. First, the present study uses a larger range of footwear thicknesses (30–60 mm versus 35–55 mm), which may explain why we do see a small, but significant, effect of thickness. Second, the firm footwear conditions in this study were purposely created with a firm EVA midsole material, whereas the footwear conditions used by Barrons et al. (2023) utilized a thermoplastic polyester elastomer that was substantially more compliant, which may play a role in how the thickness of the material affects metabolic cost. It could also be that the effect of midsole thickness on metabolic cost is nonlinear, and that the metabolically optimal thickness is at or below the thicknesses tested in these experiments. However, this metabolically optimal thickness is likely to be individualized and related to the runner's height, leg length, speed and other characteristics. From minimal footwear to footwear with a midsole thickness of ∼30 mm, larger effects of increasing midsole thickness on metabolic cost have been reported (Hébert-Losier et al., 2022), but those are likely to be related to other between-shoe differences, such as midsole material and an embedded, stiffening plate. The findings of Tung et al. (2014) suggest that the isolated effect of cushioning (barefoot running on similar materials to those used in the present study) may be metabolically optimal at 10 mm thickness, compared with 20 mm and no cushioning. However, Tung et al. (2014) purposely manipulated cushioning thickness without changing effective leg length, whereas the purpose of the present study was specifically to change effective leg length.

Although our purpose was to look at the effective leg length and energetics, we additionally quantified joint mechanics to provide further insights into the effects of midsole properties on running biomechanics and energetics. We were primarily interested in evaluating whether longer effective leg lengths would lead to longer step lengths and, if so, whether that would lead to reduced metabolic rates, or whether such longer steps would lead to increased hip extension moments in the sagittal plane, offsetting the relative metabolic savings associated with longer steps. However, there was no difference in hip extension moment at heel strike. Because there was no difference in step length, this is to be expected. Yet, there was a slight increase in positive power at the hip with increased midsole thickness at heel strike. Changes in metabolic cost of running are not easily explained by changes in joint mechanics, as there are many aspects that are not taken into account, such as power transfer from joint to joint, elastic storage and return form tendons, muscle fiber operating length and shortening velocity, different efficiencies for concentric and eccentric work, and many others (Beck et al., 2022; Bohm et al., 2023; Farris and Sawicki, 2011; Hata et al., 2024; Monte et al., 2020).

### Midsole material

In the tallest condition (60 mm), the compliant midsole led to a shorter effective leg length during midstance, but not during early and or late stance, compared with the firm midsole material. This confirms that the firm midsole resulted in a greater effective leg length during midstance than the compliant midsole did. Additionally, this provides evidence that the compliant midsole indeed achieved greater midsole compression during running.

Furthermore, the compliant midsole led to a decrease in metabolic cost compared with the firm midsole of the same thickness. It should be noted that the compliant midsole also had a greater resilience (i.e. greater energy return), which may also play a role in reducing metabolic cost (Perry et al., 2025). Because midsole compliance increased with increased midsole thickness, and running with increased midsole thickness led to a higher metabolic cost, midsole resilience may be the more important factor in reducing metabolic cost. This provides a strong indication that midsole material properties, not midsole thickness, account for the improvements in running performance seen in AFT.

### Stiffness

As midsole thickness increased in the firm material, the compression stiffness decreased. This is expected based on the viscoelastic properties of the midsole (Shorten, 2024). When midsole thickness doubled from 30 to 60 mm, the stiffness decreased from 104 to 75.6 N mm^−1^ (27.3% lower). This is due to the nonlinear stiffness of the foam materials and the simplified method of calculating compression stiffness for this study. At a force of 1800 N (greater than what may occur at the heel during running), the lower midsole thickness approaches a ‘bottoming out’ of the material, where the material no longer deforms under a force (see Fig. 1B). Additionally, the input and output energy increase, but percentage energy return (resilience) remains relatively unchanged. As expected, the shoe's compression stiffness decreases when the midsole material is changed from a firm to compliant material. The input and output energy increase, and percentage energy return is also increased. The increase in percentage energy return seems to be the only distinctive characteristic between midsole thickness effects and midsole material effects, suggesting that this is a factor in the savings in metabolic cost.

Vertical stiffness of the leg plus midsole decreased with increased midsole thickness. This decrease was small and likely to be related to an increase in midsole compression. Although the decrease in vertical stiffness is small, it may indicate that increased midsole compression led to larger center of mass displacements, rather than being counteracted by a stiffening of the leg to maintain center of mass mechanics as observed for altered surface condition (Daley and Biewener, 2006; Ferris et al., 1998, 1999). However, there was no significant effect of midsole material on vertical stiffness, which agrees with existing literature on running on different surface stiffnesses (Ferris et al., 1998; Kerdok et al., 2002).

There was no effect of midsole thickness on leg stiffness (two-dimensional stiffness of the leg plus midsole, between the center of mass and the center of pressure). However, leg stiffness was lower with the compliant material than with the firm material, contrasting with existing literature showing that overall system stiffness (i.e. leg plus surface) remains constant across different surface stiffnesses (Kerdok et al., 2002). The differing effects of midsole thickness and midsole material on vertical and leg stiffness highlight that how the body adjusts to these footwear conditions is more complex than footwear compliance alone. Additionally, this provides additional evidence that the interaction of leg and footwear stiffness differs from that of leg and surface stiffness owing to more complex structures of footwear, such as a forefoot rocker, longitudinal bending stiffness, etc. Existing evidence on the effect of running footwear stiffness on leg stiffness is conflicting, with some studies showing increased leg stiffness (i.e. stiffness from hip joint center to ankle joint center) with greater midsole cushioning (Kulmala et al., 2018) and others showing no change (Hata et al., 2022). Other estimates of stiffness (i.e. system stiffness of leg plus midsole) from accelerometer measures show no difference in leg stiffness from different midsole stiffness (Rodrigo-Carranza et al., 2024). In contrast, leg/system stiffness adjustments to changes in surface stiffness seem to be more consistent (Ferris et al., 1998, 1999; Kerdok et al., 2002), with overall system stiffness remaining constant by adjustments in leg stiffness.

### Mechanics

Sagittal plane kinetics suggest that increasing midsole thickness leads to large reductions in peak positive and negative power at the ankle, reductions in peak positive knee power and reductions in peak negative hip power, yet a small to moderate increase in positive hip power at heel strike. However, the frontal plane mechanics show increases in positive and negative ankle eversion power. Although increased ankle eversion has not been directly linked to metabolic cost (Pizzuto et al., 2019), it is unlikely to be beneficial. Moreover, frontal plane mechanics are often discussed in relation to stability (Barrons et al., 2023), and increased midsole thickness has also been linked to increased whole-body instability (Kettner et al., 2025). Based on these findings, the increase in metabolic power with increased midsole thickness may be related to frontal plane mechanics and instability, but further investigation is needed.

In the sagittal plane, the compliant midsole led to a reduction in peak positive (plantarflexion) and negative (dorsiflexion) power at the ankle, but no differences at the knee or hip. In the frontal plane, there were substantial decreases in peak joint powers at the ankle, knee and hip with the compliant material. This contrasts with the increasing frontal plane ankle power due to midsole thickness. If increased midsole thickness leads to increased instability (Barrons et al., 2023; Kettner et al., 2025), it may be that increased midsole compliance reduces the instability caused by increased midsole thickness, making the footwear better suited for long-distance running. This may be due to the reduced distance from the foot to the ground during midstance and reduced effective vertical and medio-lateral moment arms about the joints with a more compliant midsole, but further investigation would be needed to link reduced frontal plane joint powers to a metric of center of mass stability, such as a maximum Lyapunov exponent.

Increased midsole thickness led to decreased braking and propulsive impulse but not to differences in contact time. However, braking and propulsive impulse increased with the compliant material, with a corresponding increase in contact time. Hoogkamer et al. (2018, 2019) found that in AFT (greatest midsole thickness and compliance, lowest metabolic cost), participants ran with an increased braking and propulsive impulse, without a change in contact time. These findings suggest that increased braking and propulsive impulses are associated with lower metabolic cost, although the same participants were not shared between the studies. This is counter intuitive, as minimizing braking impulses would be expected to increase efficiency, as less effort is needed to compensate for energy lost during braking. However, an increase in braking and propulsive impulse may highlight improved energy storage and return in the midsole. Still, the relative magnitude of this change is small and may be due to differences in joint mechanics further up the leg.

### Effect of mass

The magnitude of the change in metabolic cost due to material (1.7% decrease from firm to compliant) was greater than the change due to midsole thickness (0.4% increase per 10 mm added). For perspective, each 100 g added to the foot increases the metabolic cost of running by ∼1% (Franz et al., 2012; Frederick et al., 1984). For these footwear conditions, the mass difference before equalizing was 154 g between the 30 mm and 60 mm firm conditions. This suggests that the estimated additional effect of added mass due to increased midsole thickness (∼1.54% increase in metabolic cost from 30 to 60 mm) would be larger than the effect due to increased thickness itself (1.14%). Therefore, when practically increasing midsole thickness (without mass matching the baseline condition), the negative effect of added midsole thickness will likely be even greater than the results presented in this study.

### Exploratory findings

Our initial findings provided us with an additional research question: if increasing the thickness of a firm midsole material leads to increased metabolic cost, does increasing the thickness of the compliant midsole change metabolic cost similarly? To answer this question, we set out to evaluate the metabolic cost of running in shoes with a 30 mm compliant midsole. The compliant material led to a 2.4% lower metabolic power than for the firm material, but there was no significant difference between the 30 mm and 60 mm compliant midsole conditions. Although our results do not provide conclusive evidence, based on the small sample size (*N*=6), they do again suggest that the effect of midsole material on metabolic cost is more important than that of thickness, when footwear mass is held constant. If footwear mass was not equalized between conditions, this conclusion could have been stronger, as the compliant material is less dense than the firm material. However, future work is needed to confirm these findings.

### Overview

In this study, we looked at the effects of midsole thickness and midsole material on effective leg length and the metabolic cost of running. There are several measures that show similar trends between the two conditions. For example, peak ankle plantarflexion decreases as midsole thickness increases, just as peak ankle plantarflexion decreases with the firm compared with the compliant material. Similarly, midsole stiffness decreases (i.e. compliance increases) as midsole thickness decreases and with the firm compared with the compliant material. However, there are some measures in which these two measures diverge. Specifically, metabolic power increases with increased midsole thickness, while metabolic power decreases with the firm compared with the compliant material. This may be explained by the greater midsole resilience with the compliant material. Additionally, frontal plane ankle joint power tends to increase for increased midsole thickness but decreases with the firm compared with the compliant material. Moreover, midsole percentage energy return from mechanical testing increases with the firm compared with the compliant material but is mostly unchanged by midsole thickness. If runners are able to benefit from the energy returned from the midsole, this may by a mechanism for improved metabolic cost in footwear with highly resilient materials (Matijevich et al., 2023).

### Limitations

This study has several limitations. Most notably, modifying one variable in footwear design will inherently affect another. For example, increasing midsole thickness will increase the longitudinal bending stiffness and the mass of the shoe. Regarding the former, all shoes in this study had a stiff plate, likely making the effect of increased midsole thickness on longitudinal bending stiffness negligible between the conditions tested in the main experiments of this study, as they were all very stiff relative to on-the-market shoes. Additionally, the thicknesses of midsoles used in this study (30 mm to 60 mm) meant that all footwear conditions had a significant forefoot rocker that may not be present in midsole thickness substantially less than 30 mm. Regarding mass, all shoes were mass matched to the heaviest condition. As a result, however, all shoe conditions were far heavier than one could find in on-the-market running shoes. This also ignores the practical effects of adding midsole thickness to shoes. In reality, increased metabolic cost from adding midsole thickness would be accompanied by an increase in metabolic cost from added mass of the added thickness. Moreover, these footwear conditions could have a different effect if the participants were able to adapt over the course of several days or weeks. However, owing to the impractical nature of the footwear conditions (mainly due to the mass), a longitudinal study may not be relevant. Inclusion of the 30 mm compliant condition in the main experiments would have provided additional insights regarding the interaction of midsole thickness and material, but because the main aim of the study focused on midsole thickness and the effects on effective leg length, and not resilience, we opted for a firm foam for our main conditions. Lastly, including a larger female cohort would have allowed us to include sex-based analysis, which may be of importance when looking at the effect of midsole thickness on metabolic cost (Mason et al., 2024). Nonetheless, this study adds to our understanding of specific features of AFT.

### Conclusions

This study tested the hypothesis that increasing the midsole thickness of a running shoe can lead to an increased effective leg length, thereby decreasing the metabolic cost of running. Despite finding that effective leg length did indeed increase, step length was unaltered, while metabolic cost increased. However, our hypothesis that increased midsole compliance for a given midsole thickness would reduce the metabolic cost of running was shown to be correct. We interpret this to indicate that the benefit seen with AFT is not due to increased effective leg length but to increased resilience of the compliant midsole. However, whether this mechanical energy can be returned to the runner has yet to be demonstrated directly.
